# LIFT: Lifting the Impacted fetal head; the Fetal Pillow^®^ and Tydeman^®^ Tube trial: protocol for a prospective observational study to evaluate two disimpaction devices

**DOI:** 10.1186/s12884-026-09191-1

**Published:** 2026-06-12

**Authors:** Laura van der Krogt, Hannah Rosen O’Sullivan, Megan Hall, Paul T Seed, Annette Briley, Graham Tydeman, Andrew Shennan

**Affiliations:** 1 Department of Women and Children’s Health, School of Life Course and Population Sciences, King’s College London, 10th Floor, North Wing, St Thomas’ Hospital, Westminster Bridge Road, London, SE1 7EH UK; 2 Caring Futures Institute, College of Nursing and Health Sciences, Flinders University, and Northern Adelaide Local Health Network, Adelaide, South Australia Australia; 3 Maternity Services, Victoria Hospital, NHS Fife, Kirkcaldy, United Kingdom

**Keywords:** Impacted fetal head, Caesarean section, In-labour caesarean section, Full dilation caesarean section, Tydeman^®^ Tube, Fetal Pillow^®^

## Abstract

**Background:**

Impacted fetal head (IFH) at full dilatation caesarean section (CS) is an increasingly recognised obstetric emergency associated with significant maternal and neonatal morbidity. Despite its importance, there is limited evidence to guide management, particularly the use of disimpaction devices.

**Methods:**

LIFT is a prospective observational trial comparing the use of two commercially available disimpaction devices—the Fetal Pillow^®^ and the Tydeman^®^ Tube —for the management of IFH at full dilatation caesarean section.

**Discussion:**

The LIFT study will compare the performance of the Fetal Pillow^®^ and Tydeman^®^ Tube in the management of IFH and contribute to the development of an evidence base to support best practice. As awareness of the clinical significance and implications of IFH continues to grow, generating robust data on available management options is essential. This study represents an important step toward optimising the safe and effective management of this challenging obstetric emergency.

**Trial registration:**

NCT07372768, registered 28/01/2026, https://clinicaltrials.gov/study/NCT07372768.

## Background

Impacted fetal head (IFH) is a recognised and important complication of emergency caesarean section (CS), especially those performed in advanced labour (7–9 cm) or at full dilatation (10 cm). It occurs when the fetal head is low and wedged in the maternal pelvis, making it challenging for the operator to deliver and ‘requires the use of additional manoeuvres and/or tocolysis to disimpact and deliver the fetal head’ [1]. It is estimated to complicate 10% of all emergency CS and 16–32% of all those a full dilatation [1].

IFH is associated with significant maternal and neonatal morbidity including uterine extensions, haemorrhage, visceral injury, sepsis, and fetal trauma such as skull and long bone fractures, hypoxic ischaemic encephalopathy and perinatal mortality [2]. A recent UK Obstetric Surveillance Survey (UKOSS) demonstrated that 3% of babies suffer severe injury or die secondary to complications of IFH and 6% of mothers require admission to intensive care units [3].

With the serious complications associated with IFH, it follows that there are significant medicolegal consequences. The UK’s National Health Service Resolution’s Early Notification Scheme identified that IFH was a contributory factor in 9% of all cases of severe brain injury, and nearly 10% of all maternity claims [4, 5]. In the UK, maternity accounts £3.5 billion of the notified claims and £1.3 billion of total clinical negligence payments in 2024/25 [6].

There is no consensus amongst clinicians on the best management technique and various have been described, including repositioning the operating Table [7], switching the delivering hand [7], administration of tocolytics [8, 9], and additional manoeuvres including vaginal push-up [10, 11] or reverse breech extraction [12]. Disimpaction devices, such as the Fetal Pillow^®^ and Tydeman^®^ Tube have also been developed [13]. There is no consensus on the optimal management of IFH and selection of manoeuvre or device is currently largely dependent on clinician experience or preference.

Disimpaction devices aim to elevate the fetal head vaginally to aid delivery, and are an alternative to the vaginal push up technique, which has been associated with increased maternal and neonatal morbidity.

The Fetal Pillow^®^ is a medical device designed to disimpact the fetal head at the time of full dilatation CS. It is a silicone balloon device that is inserted vaginally into the posterior fornix beneath the fetal head. It is inflated with up to 180mls of water, aiming to disimpact and elevate the head before starting the CS [14]. Currently it is used in the United Kingdom, the United States of America, New Zealand and Australia [15, 16].

The Tydemanâ Tube is a novel disimpaction and vacuum release device which consists of a semi-rigid wide bore silicone tube and a flexible silicone cup [17]. There are three components to the device: the cup, which is angled to fit against the fetal head and encourage the elevating force to be both superior and anterior in direction, whilst spreading force over a large area to minimise localised pressure; the tube, which serves as a handle and is hollow with a wide internal diameter, and finally the end-piece, which has a marker to ensure correct orientation during use. The device is inserted by an assistant to elevate the head by pushing it upwards, disimpacting the head from the pelvis and facilitating delivery [17]. The wide bore of the tube allows air to enter from below and ameliorate any vacuum effect. The Tydeman^®^ Tube may be inserted into the vagina in late labour from 7 cm dilatation before or during a CS, when an IFH is either anticipated or is encountered unexpectedly [17].

At present there is a paucity of evidence underlying the use of disimpaction devices [18]; therefore, the LIFT study has been developed to compare the Fetal Pillow^®^ and Tydeman^®^ Tube in the management of an IFH.

## Methods

### Aims and objectives

The aim of the LIFT trial is to compare the effectiveness of the Tydeman^®^ Tube and the Fetal Pillow^®^ for the management of an IFH at full dilatation caesarean section. The aim of the substudy is to compare the effectiveness of the Tydeman^®^ Tube with usual care for management of an IFH at CS between 7 and 9 cm dilatation.

The primary objective is to determine whether the Tydeman^®^ Tube is as effective as the Fetal Pillow^®^ for managing an IFH at full dilatation CS, by comparing time from uterine incision to delivery after using the devices.

The secondary objectives are:

- to compare maternal and neonatal outcomes following the use of the Tydeman^®^ Tube or Fetal Pillow^®^ in the main study, and between the Tydeman^®^ Tube and control group in the substudy
- to evaluate the acceptability of both devices to women and clinicians

### Design

This is a prospective non-inferiority observational comparison trial with two sequential interventional arms, the first using the Fetal Pillow^®^ and the second using the Tydeman^®^ Tube. Each device has regulatory approval for routine use and therefore can be introduced into clinical practice.

At trial commencement, obstetric and midwifery staff will be trained to use the Fetal Pillow^®^ by members of the research team during dedicated training sessions. The Fetal Pillow^®^ will then be introduced into clinical practice for 3–6 months for use during all full dilatation CS, or until the recruitment target is reached (*n* = 30). The Fetal Pillow^®^ will be inserted and inflated prior to full dilatation CS, in all eligible women. Consent will be sought retrospectively, see ‘Patient Recruitment’. Once the recruitment target for the Fetal Pillow^®^ has been reached, the device will be withdrawn from clinical areas and will no longer be available for use.

Obstetric and midwifery staff will then be trained to use the Tydeman^®^ Tube and the above process repeated over a 3–6-month period, or until the recruitment target is reached (*n* = 30). For eligible women, the Tydeman^®^ Tube will be inserted prior to commencement of all full dilatation CS and will be used to elevate the fetal head if felt to be required during the caesarean by the operating surgeon.

Should recruitment be reached in a shorter time frame than expected then each arm will continue for the set time frame (3–6 months) to allow for increased data collection and loss to follow up, to a maximum of 40 women per arm.

During the Tydeman^®^ Tube arm of the study, additional Tydeman^®^ Tubes will be available for clinicians to use at their discretion for caesarean sections performed at 7–9 cm cervical dilatation as per the manufacturer’s Instructions for use, which stipulates that the Tydeman^®^ Tube can be used from 7 to 10 cm. If, during a caesarean section, the operating surgeon unexpectedly encounters an IFH they can request a Tydeman^®^ Tube to use. Consent will be sought retrospectively as per the rest of the trial. This cohort will be in addition to the recruitment target of 30 women for each device. Outcome data will be compared with an historical control group of standard practice (using no device).

Recruited participants and clinicians involved in device use will be asked to complete a questionnaire regarding their views of the device.

### Setting

The LIFT trial will be conducted at St Thomas’ Hospital, a tertiary level maternity unit in South London, UK. Neither device is currently in use at this site.

### Population

Main study; women requiring a CS at full cervical dilatation. Substudy; clincian request for a Tydeman Tube for women found to have an IFH at CS between 7 and 9 cm dilatation.

### Inclusion and exclusion criteria

#### Inclusion criteria

For main study:

- Women undergoing full dilatation caesarean section
- Singleton pregnancies
- Cephalic presentation
- Aged > 16 years old
- ≥ 37 weeks’ gestation

For sub-study:

- Women undergoing caesarean section at 7–9 cm where IFH is encountered
- Singleton pregnancies
- Cephalic presentation
- Aged > 16 years old
- ≥ 37 weeks’ gestation

For clinician sub-cohort:

- Operating surgeon/surgeons or operator of device
- Willing to complete questionnaire

#### Exclusion criteria

For main study:

- Allergy to silicone rubber
- Major congenital abnormalities
- Major anomaly of the fetal head (i.e. large encephalocele)
- Intrauterine death
- Suspected chorioamnionitis
- Active genital infection inc. Herpes Simplex Virus

For sub-study:

- Allergy to silicone rubber
- Major congenital abnormalities
- Major anomaly of the fetal head (i.e. large encephalocele)
- Intrauterine death
- Suspected chorioamnionitis
- Active genital infection inc. Herpes Simplex Virus

For clinician sub-cohort:

- Clinician not directly involved in use of device or operation

### Trial flowchart

**Fig. 1:**
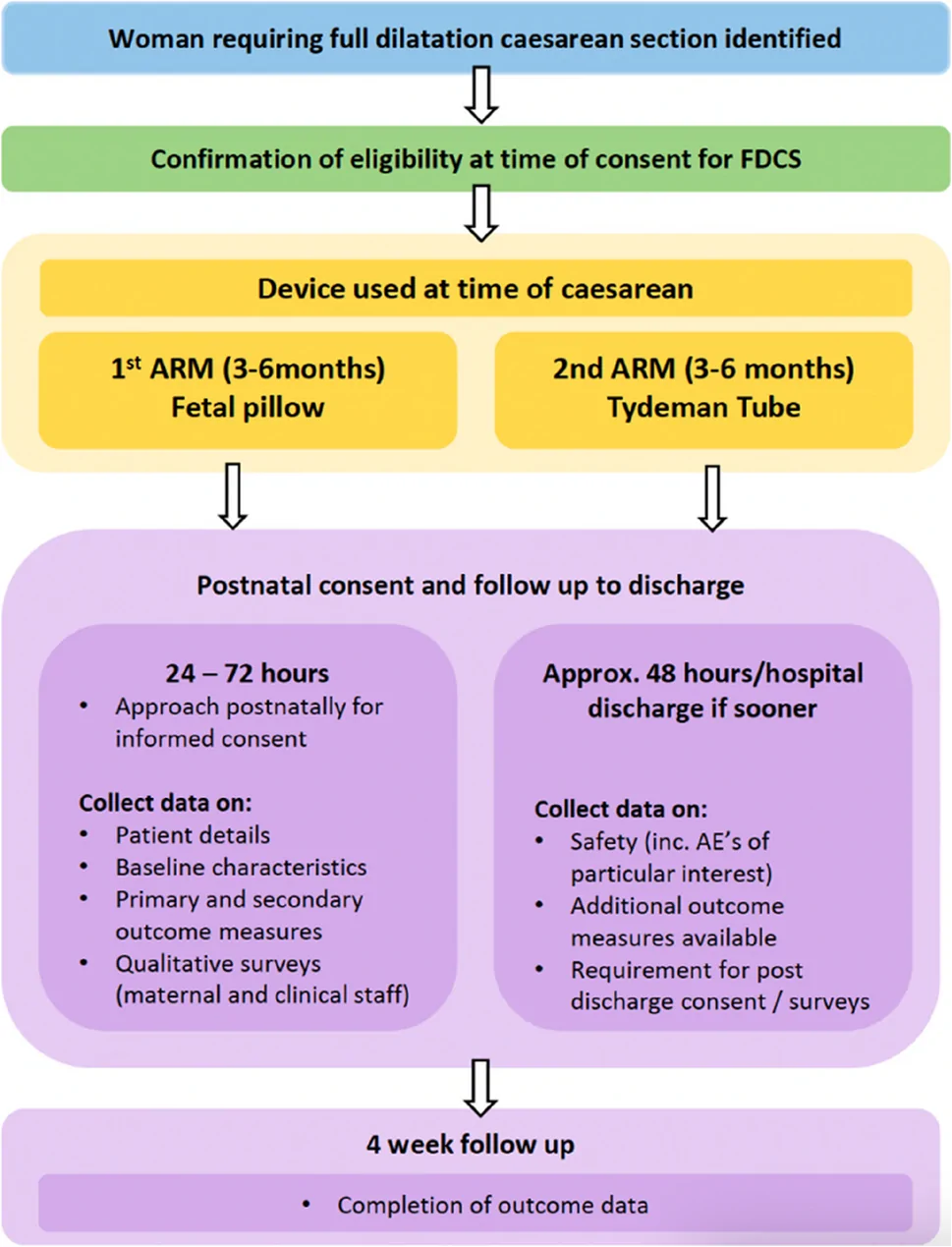
Trial flowchart

Figure 1 shows the trial flowchart outlining the study pathway.

### Patient recruitment

A full dilatation caesarean section is a common and unpredictable clinical emergency, and there is no consensus of how to predict in which cases an IFH will occur. Around 20% of all deliveries are by emergency caesarean section, and 5–7% of these are performed at full dilatation. In usual clinical practice, use of manoeuvres and/or disimpaction devices would be at the discretion of the operating team. We have designed the recruitment and consent process as per the protocols of similar established emergency obstetric trials (for example the COPE trial) [19].

**Fig. 2:**
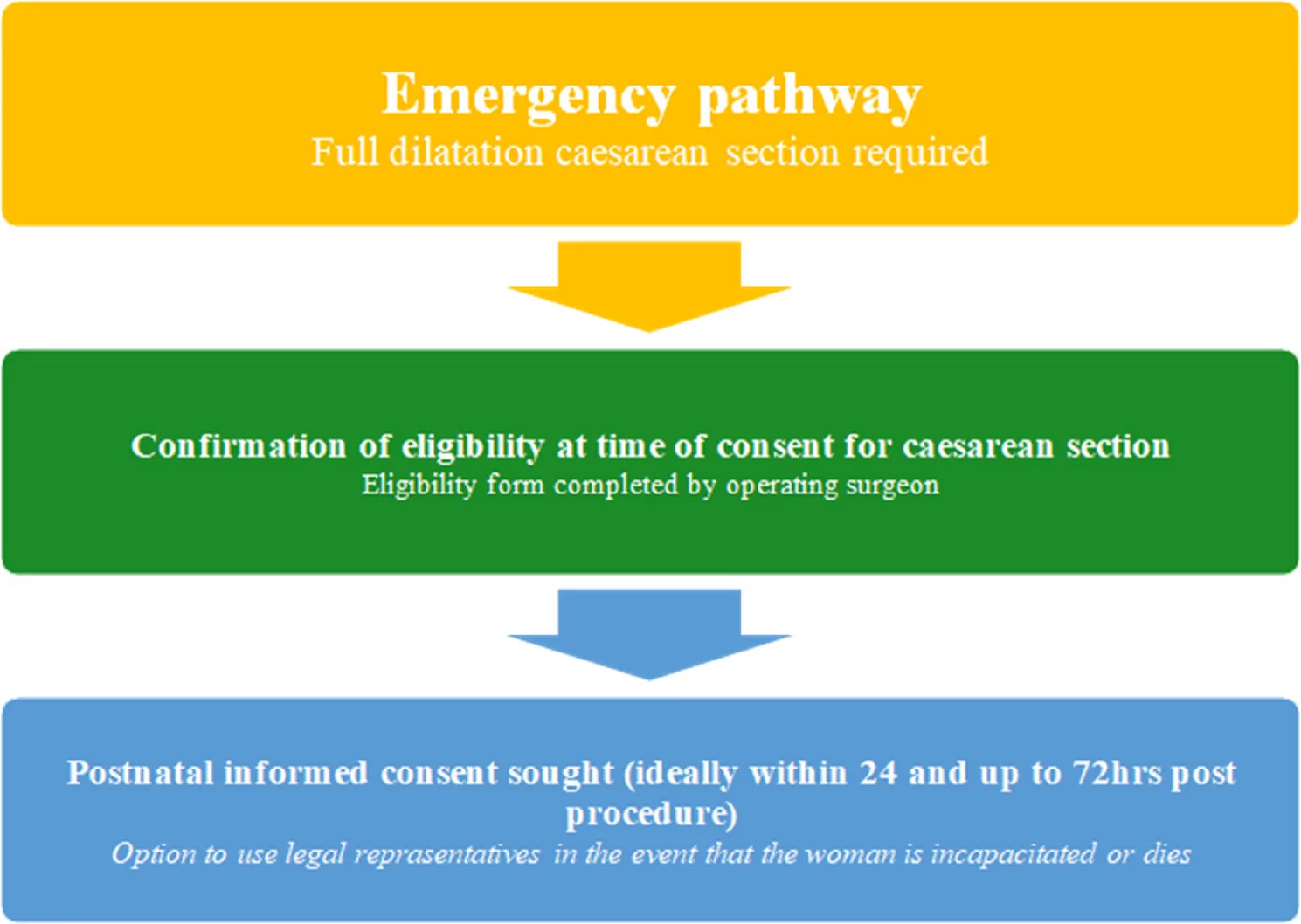
LIFT recruitment via emergency pathway

LIFT participants will be recruited via the following emergency pathway, see Fig. 2.

Use of the Tydeman Tube or Fetal Pillow during full dilatation caesarean section will occur if eligibility criteria is met at time of consent for caesarean section. Postnatally, once the woman is stable and ideally within 24 hours (but potentially up to 72 hours, or prior to transfer to another hospital/discharge), full study information will be provided to the woman. Before approaching participants, the research team will ensure this is appropriate timing with a member of the clinical team. A patient information leaflet will be provided and if the patient is willing to participate, permission will be sought for disclosure of identifiable data, and the woman will be asked to sign the trial consent form.

Should a participant not regain capacity to consent within 72 hours, full study information should be provided as soon as possible to her Personal Consultee or Nominated Consultee (should a Personal Consultee not be available). The Personal or Nominated Consultee will be asked to sign the Personal or Nominated Consultee Declaration Form. Should a woman regain capacity to consent after this time and before 4 weeks postnatally, then full study information will be provided to them, and written informed consent will be sought should they wish to participate in the study.

Consent is expected to be sought for all women prior to discharge or transfer, however if this is not enacted, postal or electronic consent should be sought no more than 4 weeks after the event. Women should be contacted by trial staff within 5 working days to inform them of their involvement and provide details of the trial. If the woman is not willing to provide consent, her details will be withdrawn. If the woman is willing to provide consent, a patient information leaflet and consent or electronic consent form will be sent via post or email to the address provided by the woman, depending on their preference.

Participation in the study is voluntary and participants are able to withdraw their consent at any time. This option will be provided within the written information on the participant information sheet and discussed at the time of informed consent. The participant will be told that withdrawal at any time would not affect their clinical care. If consent is withdrawn then any outcome data collected will not be used for analysis and the participant will be removed from the study. If consent for data storage and analysis is withdrawn at any time the participants data will be withdrawn from the analysis.

### Outcome measures

#### Primary outcome

Time from uterine incision to delivery of baby (seconds).

#### Secondary outcomes

Maternal outcomes:

- Uterine incision to closure time (minutes)
- Estimated blood loss
- Need for blood products
- Volume of blood products required
- Uterine angle extensions
- Visceral injury (ureter, cervix, bladder, bowel)
- Hysterectomy
- Genital tract trauma
- Maternal admission to higher level care (HDU/ITU)
- Maternal death
- Length of stay (nights)
- Post-operative wound infection
- Endometritis
- Change in haemoglobin pre and post procedure

Neonatal outcomes:

- Birth trauma (including, but not limited to, intracranial haemorrhage, skull fracture, other fracture, facial injury including bruising/facial nerve palsy, any other nerve injury)
- Apgar scores at 5 and 10 min
- Need for resuscitation
- Admission to higher level care (SBCU/NICU)
- Length of stay in higher level care (up to 4 weeks)
- Arterial cord pH
- Hypoxic ischaemic encephalopathy (grades 1, 2 and 3)
- Perinatal death
- Neonatal feeding

Qualitative clinician outcomes using questionnaire:

- Evaluation of the Tydeman^®^ Tube and Fetal Pillow^®^ using Likert scale (acceptability, ease of use)
- Perception of impacted head at time of caesarean section
- Perception of ease of delivery using each device
- Need for elevation of the fetal head using Tydeman^®^ Tube

Qualitative maternal outcomes using questionnaire:

- Acceptability of Tydeman^®^ Tube and Fetal Pillow^®^

### Data handling and storage

Participants will be allocated a unique study ID number at time of recruitment, with no storage of patient identifiable information. To ensure confidentiality and data safety, all patient data will be link-anonymised at time of data entry in accordance with the Data Protection Act 2018 and UK GDPR directive. All maternal and neonatal outcome data will be collected as part of routine clinical care, including demographic and clinical data.

Data will be stored on a RedCap database. This is a secure third-party cloud provider. The database is very clear and simple to use, and predefined validation rules and data monitoring facility help to ensure accurate data entry. Access to the database will be managed by the RedCap team in collaboration with the Principal Investigator. Prospective new users must demonstrate compliance with legal, data protection and ethical guidelines before any data are released.

### Data retention and sharing

Any data relating to pregnancy care is required to be stored for at least 25 years. At the end of the study, pseudonymised research data will be locked and downloaded from the LIFT Database (on RedCap), saved in a read only format and archived according to Sponsor requirements (with Iron Mountain). Copies will also be kept on KCL Sharepoint. A file note will be placed in the Investigator Site File detailing the location of electronic data.

The consent form states that other researchers or commercial partners may wish to access anonymised data in the future. Should this be a research group outside of the trust or a commercial partner, data will be shared only according to GSTT/KCL data governance policies and where appropriate, a data agreement would be put in place when required.

### Data analysis, sample size and power calculations

Time between uterine incision and delivery of the baby reflects the degree of difficulty in disimpacting the fetal head and will be used as the primary outcome measure to compare efficacy of the devices. Estimation of usual time from uterine incision to delivery was obtained from a robust clinical database capturing all maternity statistics from our unit over a period of one year. Sample size calculation was based on an observed mean time from uterine incision to delivery of 97 s (standard deviation 94 s).

To demonstrate that the Tydeman^®^ Tube is non-inferior to the Fetal Pillow^®^, non-inferiority was defined as an increase in uterine incision to delivery time of no more than 97 s (corresponding to a doubling of mean time to 194 s). We chose doubling of the time from uterine incision to delivery as a clinically important outcome, as this would take the average time to over 3 min. A delivery time of over 3 min at caesarean section has been associated with the development of hypoxic ischaemic encephalopathy, hence exceeding this is non-desirable.

30 participants per arm provides 95% power (one-sided alpha 0.05) to determine non-inferiority. We therefore aim to recruit 30 participants to each arm as minimum. If the recruitment target is reached sooner than expected (i.e. within 3 months) then recruitment will continue up to 40 participants per arm for a maximum of 6 months. In the substudy the Tydeman^®^ Tube will only be used when an IFH is encountered unexpectedly. We do not have data to support how frequently this may occur during the study period and this will be an observational arm without pre-defined sample size.

### Trial monitoring

The Chief Investigator will be responsible for the ongoing management of the study. The Sponsor will monitor and conduct audits on a selection of studies in its clinical research portfolio. Monitoring and auditing will be conducted in accordance with the UK Policy Framework for Health and Social Care and in accordance with the Sponsor’s monitoring and audit procedures.

### Assessment of safety

The Data Monitoring Committee (DMC) will be convened to ensure the wellbeing of study participants and the quality of the data collected. The committee will periodically review study progress and outcomes as well as reports of serious adverse events (SAEs). The DMC will, if appropriate, make recommendations regarding the continuance of the study or modification of the study protocol.

#### Procedures for recording and reporting adverse events

Serious adverse Event (SAE) or Serious Adverse Reaction (SAR) is:

Any adverse event, adverse reaction or unexpected adverse reaction, respectively, that.

- Results in death
- Is life-threatening
- Required hospitalisation or prolongation of existing hospitalisation, excluding admission for birth or unrelated pregnancy condition
- Results in persistent or significant disability or incapacity
- Consists of a congenital anomaly or birth defect
- Is otherwise considered medically significant by the principal investigator

Expected serious adverse events are those events which are expected in the patient population or as a result of the routine care/treatment of a patient. Full dilatation caesarean section is known to be associated with certain risks and performed routinely in clinical practice. Ashis study is evaluating a new device and therefore any serious adverse events (SAEs) and serious adverse reactions (SARs) which are believed to be related to device use rather than to full dilatation caesarean section will be reported as SAEs, including;

Maternal:

- Major obstetric haemorrhage (≥ 1500mls)
- Organ injury
- Evidence of uterine rupture
- Hysterectomy
- Maternal ITU admission/death

Fetal:

- Low Apgar scores (≤ 3 at 5 min)
- Arterial cord pH < 7
- Admission to NICU/SCBU
- HIE grades 1, 2 and 3
- Skull fractures

The following common full dilatation caesreaen section complications will not be considered SAEs:

- Hospitalisation > 48 hours
- Postpartum haemorrhage < 1500mls

### Reporting responsibilities

All SAEs (except those specified in this protocol as not requiring reporting) will be actively monitored and reported from the time of procedure and up to hospital discharge or 4 weeks, whichever is sooner. Identified SAE’s will be reported by the Investigator to the GSTT R&D Office and CI for review. Only reports of Serious Adverse Events (SAEs) that are: related to the study (i.e. they resulted from administration of any of the research procedures) and unexpected (i.e. not listed in the protocol as an expected occurrence) should be reported to the REC.

Medical judgement should be exercised by the trial investigator in deciding whether an AE or AR is serious in other situations. Important AEs or ARs that are not immediately life threatening nor result in death or hospitalisation but may jeopardise the subject or may require intervention to prevent one of the outcomes listed in the definition above, should also be considered serious.

Non-serious adverse events or reactions will also be collected and recorded.

### Protocol amendments

Any changes in research activity, except those necessary to remove an apparent, immediate hazard to the participant, must be reviewed and approved by the Chief Investigator and the Co-Sponsors notified. Substantial amendments to the protocol must be submitted in writing to the appropriate REC, Regulatory Authority and local R&D for approval prior to participants being enrolled into an amended protocol.

### Ethical approval

This study has been reviewed and approved by Yorkshire & The Humber - Bradford Leeds Research Ethics Committee. REC Reference Number 25/YH/014.

### Insurance/indemnity

The study is co-sponsored by King’s College London (KCL) and Guys and St Thomas’ NHS Foundation Trust (GSTT). The sponsors will, at all times, maintain adequate insurance in relation to the study. KCL through its’ own professional indemnity (Clinical Trials) & no-fault compensation and the GSTT having a duty of care to patients via NHS indemnity cover, in respect of any claims arising as a result of negligence by its employees, brought by or on behalf of a study participant.

### Sponsors

This study is co-sponsored by King’s College London Guys and St Thomas’ NHS Foundation Trust.

### Funding

This study is funded through Guys and St Thomas’ Charity (reference number: TCF 190908). Rocket Medical are providing Tydeman Tubes and funding for Fetal Pillows.

### Publication and dissemination policy

The trial management group will be responsible for the dissemination and publication policy. Results will be disseminated via publication in high quality research journals and via conference presentations.

## Discussion

With growing recognition of the clinical importance of IFH, and the need to establish appropriate training and optimise techniques to improve patient outcomes, the LIFT study is timely. Its findings will strengthen the evidence base and evaluate the introduction disimpaction devices into routine clinical practice.

### Trial status

Protocol version 1.1, 22/07/2025.

Recruitment due to begin March 2026 and continue until March 2027.

## Data Availability

Not applicable. This paper describes a study protocol and therefore contains no data.

## Abbreviations

CS: Caesarean section
HDU: High dependency unit
IFH: Impacted fetal head
ITU: Intensive care unit
NICU: Neonatal intensive care unit
REC: Research ethics committee
R&D: Research & Development
SCBU: Special care baby unit
